# Altered brain connectivity in mild cognitive impairment is linked to elevated tau and phosphorylated tau, but not to GAP-43 and Amyloid-β measurements: a resting-state fMRI study

**DOI:** 10.1186/s13041-024-01136-z

**Published:** 2024-08-30

**Authors:** Mohammad Sadeghi, Ali Azargoonjahromi, Hamide Nasiri, Arash Yaghoobi, Maryam Sadeghi, Seyedeh Saeideh Chavoshi, Shilan Baghaeikia, Nastaran Mahzari, Arina Valipour, Romina Razeghi Oskouei, Farshad Shahkarami, Fatemeh Amiri, Mahsa Mayeli

**Affiliations:** 1 School of Rehabilitation, Shiraz University of Medical Sciences, Shiraz, Iran; 2 Shiraz University of Medical Sciences, Shiraz, Iran; 3 School of Medicine, Zanjan University of Medical Sciences, Zanjan, Iran; 4 School of Medicine, Tehran University of Medical Sciences, Tehran, Iran; 5 Department of Nuclear Medicine, Children Medical Center Hospital, Tehran University of Medical Sciences, Tehran, Iran; 6 Imam Khomeini International University, Qazvin, Iran; 7 Faculty of the Veterinary Medicine, University of Tehran, Tehran, Iran; 8 Department of Pharmacy, School of Pharmacy, International Campus, Tehran University of Medical Sciences, Tehran, Iran; 9 School of Allied Medical Sciences, Tehran University of Medical Sciences, Tehran, Iran; 10 Department of clinical laboratory sciences, Qazvin University of medical sciences, Qazvin, Iran; 11 Department of Internal Medicine, School of Medicine, Tehran University of Medical Sciences, Tehran, Iran; 12 Student Research Committee, Afzalipour Faculty of Medicine, Kerman University of Medical Sciences, Kerman, Iran

**Keywords:** Mild cognitive impairment, GAP-43, Brain connectivity, Amyloid β, Tau protein, Default mode network

## Abstract

Mild Cognitive Impairment (MCI) is a neurological condition characterized by a noticeable decline in cognitive abilities that falls between normal aging and dementia. Along with some biomarkers like GAP-43, Aβ, tau, and P-tau, brain activity and connectivity are ascribed to MCI; however, the link between brain connectivity changes and such biomarkers in MCI is still being investigated. This study explores the relationship between biomarkers like GAP-43, Aβ, tau, and P-tau, and brain connectivity. We enrolled 25 Participants with normal cognitive function and 23 patients with MCI. Levels of GAP-43, Aβ1–42, t-tau, and p-tau181p in the CSF were measured, and functional connectivity measures including ROI-to-voxel (RV) correlations and the DMN RV-ratio were extracted from the resting-state fMRI data. *P*-values below 0.05 were considered significant. The results showed that in CN individuals, higher connectivity within the both anterior default mode network (aDMN) and posterior DMN (pDMN) was associated with higher levels of the biomarker GAP-43. In contrast, MCI individuals showed significant negative correlations between DMN connectivity and levels of tau and P-tau. Notably, no significant correlations were found between Aβ levels and connectivity measures in either group. These findings suggest that elevated levels of GAP-43 indicate increased functional connectivity in aDMN and pDMN. Conversely, elevated levels of tau and p-tau can disrupt connectivity through various mechanisms. Thus, the accumulation of tau and p-tau can lead to impaired neuronal connectivity, contributing to cognitive decline.

## Introduction

Mild cognitive impairment (MCI) is a common progressive neurological condition with a prevalence of approximately 21.2% among older adults residing in nursing homes and 15.56% among community-dwelling individuals aged 50 years and older worldwide [1]. The progression rate from MCI to dementia varies among studies, with an average annual rate of 10–15% [2–6]. Over a span of 6 years, more than 80% of individuals with MCI have been observed to eventually develop dementia [7].

Various biomarkers have been shown to predict MCI and Alzheimer’s disease (AD) and the chances of conversion from MCI to AD years before clinical manifestations [8–10]. Growth-associated Protein 43 (GAP-43) is a synaptic protein that plays a role in neural development, particularly in the growth and remodeling of neuronal connections [11]. Several studies have explored the possible association between elevated GAP-43 levels and cognitive decline, including in individuals with MCI [12–16]. However, the findings have been inconclusive, showing no statistically significant differences between GAP-43 measurements in MCI and cognitively normal (CN) individuals [17–19]; notably, potential disruptions in functional connectivity in the anterior default mode network (aDMN) and posterior DMN (pDMN) associated with GAP-43 have not been investigated thus far.

Amyloid beta (Aβ), tau, and P-tau are proteins that play a significant role in the development of cognitive decline like MCI. While some studies have observed increased levels of Aβ and the presence of abnormal tau proteins in certain cases of MCI [20–26], the significance of these associations with aDMN and pDMN in MCI cases remains unclear. Indeed, the role of Aβ and tau in patients with MCI requires further investigation to fully understand their relationship and implications in the development of the condition.

The default mode network (DMN) is one of the most investigated networks in AD research and has been shown to be affected in the Alzheimer’s dementia continuum. DMN encompasses interconnected regions in the medial prefrontal cortex, posterior cingulate cortex, and bilateral inferior parietal lobes and plays a role in cognitive functions such as self-referential thinking, introspection, mentalizing, and mind-wandering [27]. Disruptions in the DMN have been observed in various neurological and psychiatric conditions including MCI, AD, depression, schizophrenia, and ADHD [28, 29], and ongoing research aims to further our understanding of the DMN and its implications in these conditions. Specifically, in the context of MCI, studies utilizing rs-fMRI have revealed changes in connectivity patterns within the DMN among individuals with MCI, subjective cognitive decline (SD), early and late stages of MCI, and AD. These changes included increased functional connectivity in various brain regions such as the precuneus, anterior cingulate, angular gyrus, medial frontal gyrus, and parahippocampus. Covariance analysis further delineated differences in functional connectivity between different groups, indicating shared pathomechanisms between MCI and SD and suggesting a maladaptive short-term mechanism in maintaining cognition in LMCI and AD [30, 31].

Although the alterations of DMN in AD continuum have been formerly investigated, the potential associations between AD CSF biomarkers of synaptic growth and Aβ and tau accumulations have not been evaluated. In particular, due to the role of GAP-43 in synaptogenesis, we aimed to investigate potential patterns of alterations of DMN in response to these biomarkers and to compare these associations between CN individuals and patients with MCI.

## Methods and materials

The data for this article were sourced from the Alzheimer’s Disease Neuroimaging Initiative (ADNI) database (http://adni.loni.usc.edu), a public-private partnership launched in 2003 under the leadership of Principal Investigator Dr. Michael W. Weiner. ADNI’s primary goal is to determine whether a combination of serial magnetic resonance imaging (MRI), positron emission tomography (PET), various biological markers, and clinical and neuropsychological assessments can effectively measure the progression of MCI and early AD. Participants, aged 55 to 90, were required to undergo neuroimaging, lumbar punctures, and longitudinal follow-ups, with specific inclusion and exclusion criteria detailed elsewhere. Key exclusion criteria included a Hachinski Ischemic Score above 4, use of unapproved medications, recent changes in permitted medications, a Geriatric Depression Scale score of 6 or higher, and less than six years of education or equivalent work experience. Participants were categorized into CN, MCI, and AD groups based on ADNI’s clinical classification criteria, as detailed in other publications.

### Cognitive assessment

The Mini Mental State Examination (MMSE) is a crucial tool for assessing cognitive function in older adults, aiding in the early detection of changes in physiological status, learning abilities, and treatment responses. Since its validation in 1975, the MMSE has been widely recognized for its reliability in screening cognitive impairment. Comprising 11 questions that evaluate orientation, registration, attention and calculation, recall, and language, the MMSE has a maximum score of 30. Quick and practical, it takes only 5–10 min to administer, making it ideal for routine use across community, hospital, and institutional settings. A score of 24 or more indicates normal cognition, while scores below 24 indicate varying levels of cognitive impairment: mild (19–23), moderate (10–18), and severe (9 or lower) impairment [32–34].

### CSF measurements

CSF GAP-43 levels were measured using an in-house ELISA method. This ELISA employed a mouse monoclonal GAP-43 antibody (NM4) from Fujirebio (Ghent, Belgium) as the coating antibody, and a polyclonal GAP-43 antibody (ABB-135) from Nordic Biosite (Täby, Sweden) as the detector antibody, both targeting the C-terminal of GAP-43. Certified laboratory technicians conducted the analyses, reporting results in pg/mL within a range of 312 − 20,000 pg/mL, based on 1,268 data points. Quality control samples (QC1 and QC2) from the Clinical Neurochemistry Laboratory at Sahlgrenska University Hospital (Mölndal, Sweden) were used. During clinical evaluation, the repeatability coefficient of variation (CV%) was 5.5% for QC1 and 11% for QC2, with inter-assay CV% at 6.9% and 15.6%, respectively.

416 CSF ADNI baseline samples were assessed for Aβ1–42, t-tau, and p-tau181p levels using the multiplex xMAP Luminex platform (Luminex Corp, Austin, TX). This was facilitated by Innogenetics (INNO-BIA AlzBio3; Ghent, Belgium) immunoassay kit-based reagents designed for research use only. The immunoassay reagents included capture monoclonal antibodies and detector antibodies tailored to each biomarker, affixed to color-coded beads. Calibration curves were generated using buffered solutions with varying biomarker concentrations. Prior to analyzing ADNI and autopsy-based CSF samples, an interlaboratory study ensured assay performance and reproducibility, demonstrating less than 10% variation for CSF pool samples and less than 7% for quality controls. Further validation was conducted through test-retest analyses of 29 randomly selected samples, exhibiting high correlation coefficients (r2 values) for t-tau, Aβ1–42, and p-tau181p (0.98, 0.90, and 0.85, respectively) [35, 36]. Summary of these studies is available at http://www.adni-info.org/.

### Neuroimaging processing

Image preprocessing utilized several tools: SPM5 software (http://www.fil.ion.ucl.ac.uk/spm/software/spm5/), REST (Resting-State fMRI Data Analysis Toolkit) v1.5 (http://www.restfmri.net), DPARSF (Data Processing Assistant for Resting-State fMRI) v2.0 (http://www.restfmri.net), and MATLAB v7.11. The process involved discarding the initial 3 volumes, correcting slice time, realignment, normalization to the SPM5 EPI template, smoothing using a 4 mm Gaussian kernel, linear detrending, and bandpass filtering within the 0.01–0.08 Hz range. Linear regression was applied to correct for motion and the principal components of white matter and cerebral spinal fluid time courses.

Using group independent component analysis (GICA), a functional atlas was created, identifying 68 functional regions sorted by network, location, meta-analysis, and modularity. BOLD signals were extracted from midline anterior DMN (aDMN) and posterior DMN (pDMN) regions of interest (ROIs), merging the right and left sides into bilateral regions.

The average time course within each ROI was then correlated with every voxel in the ROI using Pearson’s correlation coefficient. This was followed by a Fisher r-to-z transformation, and the median z(r) value within each ROI, called the ROI-to-voxel (RV) correlation, was obtained. The RV correlation for the aDMN was divided by that of the pDMN to derive the DMN RV-ratio, which correlated strongly (*r* = 0.81) with coherence-based ReHo calculations of the aDMN-to-pDMN ratio.

### Statistical analysis

The data analysis was carried out in Python. The Shapiro-Wilk test was employed to test for normality. Normally distributed continuous variables are presented as mean (SD), while non-normally distributed variables are reported with median (IQR). Categorical variables are reported as frequencies. Independent sample t-tests were used to compare normally distributed continuous variables, and the Mann-Whitney U test was applied for non-normally distributed variables. Partial correlation analysis was used to examine the associations between biomarker levels and rs-fMRI-derived connectivity measures. Additionally, general linear regression models were applied for each group (CN/MCI) to explore the predictive power of biomarker values on rs-fMRI-derived metrics, controlling for age and gender. *P*-values below 0.05 were considered significant, and the Benjamini-Hochberg method was used to correct for multiple comparisons. All *p*-values reported in correlation and linear regression results are FDR-corrected.

## Results

Participants were classified into two groups: CN (25) and MCI (23). The CN group had an average age of 70.62 years, while the MCI group had an average age of 72.77 years. Educational attainment differed significantly between the groups, with the CN group averaging 18 years of education compared to 16 years for the MCI group. Gender distribution showed no significant difference, with the CN group consisting of 15 females and 10 males, and the MCI group having 11 females and 12 males. Table 1 presents the demographic characteristics of the study population. The MCI group exhibited significantly lower MMSE scores and education levels (*P* = 0.001 and *P* = 0.019, respectively). However, the mean age and gender distribution did not significantly differ between the two groups.

**Table 1:** Characteristic data of the participants

	CN (*n* = 25)	MCI (*n* = 23)	*P* value
Age, year [Mean (SD)]	70.62 (5.34)	72.77 (5.75)	0.195
MMSE [Median (IQR)]	29 (29, 30)	28 (26, 29)	0.001
Education, year [Median (IQR)]	18 (16, 19)	16 (13.5, 18)	0.019
Gender (F/M)	15/ 10	11/ 12	0.578

CN: cognitively normal; MCI: mild cognitive impairment; MMSE: Mini-Mental State Exam; F/M: female/male

Within the CN, a significant correlation emerged between average to posterior connectivity and GAP-43 (*r* = 0.491). However, we observed no significant correlation between Aβ and any of the imaging metrics in either group. Turning our attention to patients with MCI, intriguing patterns emerged. Both average to posterior connectivity and average to ventral connectivity exhibited significant negative correlations with tau (*r*=-0.59, *r*=-0.544, respectively). Additionally, we observed intriguing reverse correlations between P-tau and average to posterior connectivity, average to ventral connectivity, and Median ventral in the MCI group (*r*=-0.582, *r*=-0.553, *r*=-0.477, respectively).

In short, in CN, higher connectivity is associated with higher GAP-43 biomarker levels, while in MCI, higher connectivity is associated with lower levels of GAP-43, Tau, and P tau (Table 2).

**Table 2:** Correlation analysis between resting state fMRI weighted default mode networks and biomarkers

	GAP-43		Amyloid β		Tau		*P* tau	
	CN	MCI	CN	MCI	CN	MCI	CN	MCI
anterior to average	0.443	-0.225	0.147	0.106	0.316	-0.344	0.251	-0.320
anterior to posterior	0.381	-0.065	0.130	-0.117	0.247	-0.308	0.176	-0.271
anterior to ventral	0.137	0.117	0.050	0.004	0.148	-0.093	0.102	-0.082
average to posterior	0.491*	-0.263	0.056	-0.039	0.264	-0.590**	0.218	-0.582***
average to ventral	0.204	-0.226	-0.032	0.153	0.169	-0.544*	0.188	-0.553***
posterior to ventral	0.223	-0.147	0.103	-0.100	0.365	-0.097	0.350	-0.062
Median posterior	-0.003	0.179	0.229	0.111	-0.118	-0.125	-0.150	-0.093
Median anterior-posterior	0.100	-0.192	-0.147	-0.019	-0.061	0.052	-0.015	0.072
Median average	-0.090	0.086	0.252	0.158	-0.233	-0.035	-0.220	-0.033
Median ventral	0.237	-0.225	0.083	0.160	0.088	-0.417	-0.041	-0.477*

CN: cognitively normal; MCI: mild cognitive impairment; GAP-43: growth associated protein 43; P tau: phosphorylated Tau; * *p* value < 0.05; ** *p* value < 0.01; *** *p* value < 0.001. All *p* values are FDR adjusted. The bold values indicate significance based on *p*-value

Performing linear regression models for each study group, we assessed whether GAP-43 values could predict fMRI-derived metrics while covarying for the age and sex of participants. In the CN group, GAP-43 was found to be significantly associated with average to posterior connectivity (R^2^ = 0.25, *P* = 0.05). However, no significant associations were found in the MCI group, though trends indicate possible negative relationships between connectivity and GAP-43 levels. (see Table 3).

**Table 3:** Linear regression analysis of resting state fMRI weighted default mode networks and GAP-43 adjusted for age and gender

	CN (*n* = 25)			MCI (*n* = 23)		
	R^2^	Standardized β	*P* value	R^2^	Standardized β	*P* value
anterior to average	0.215	0.438	0.103	0.203	-0.224	0.327
anterior to posterior	0.181	0.374	0.218	0.060	-0.068	0.944
anterior to ventral	0.039	0.136	0.799	0.025	0.127	0.748
average to posterior	**0.250**	**0.489**	**0.050**	0.131	-0.276	0.547
average to ventral	0.042	0.204	0.985	0.090	-0.241	0.683
posterior to ventral	0.062	0.223	0.786	0.106	-0.153	0.786
Median posterior	0.022	-0.003	0.991	0.157	0.182	0.438
Median anterior-posterior	0.154	0.092	0.686	0.039	-0.208	0.953
Median average	0.108	-0.085	0.684	0.115	0.089	0.710
Median ventral	0.082	0.234	0.722	0.104	-0.238	0.572

CN: cognitively normal; MCI: mild cognitive impairment. All *p* values are FDR adjusted. The bold values indicate significance

Overall, these findings suggest that while higher connectivity within specific brain regions is associated with elevated levels of GAP-43 protein in CN individuals, this relationship is not observed in those with MCI, indicating potential alterations in the neurobiological mechanisms underlying brain connectivity in cognitive decline.

In addition, the results of linear regression analyses investigating the relationship between rs-fMRI measures and levels of Aβ, adjusted for age and gender, show that no statistically significant relationships are observed between any specific rs-fMRI measure and Aβ levels in either group. (Table 4)

**Table 4:** Linear regression analysis of resting state fMRI weighted default mode networks and amyloid β adjusted for age and gender

	CN (*n* = 25)			MCI (*n* = 23)		
	R^2^	Standardized β	*P* value	R^2^	Standardized β	*P* value
anterior to average	0.045	0.145	0.604	0.170	0.099	0.646
anterior to posterior	0.058	0.127	0.811	0.069	-0.115	0.920
anterior to ventral	0.023	0.050	0.821	0.012	0.004	0.986
average to posterior	0.015	0.056	0.933	0.068	-0.038	0.866
average to ventral	0.001	-0.032	0.956	0.063	0.151	0.763
posterior to ventral	0.023	0.103	0.855	0.096	-0.096	0.988
Median posterior	0.074	0.227	0.869	0.139	0.105	0.632
Median anterior-posterior	0.164	-0.136	0.682	0.003	-0.019	0.934
Median average	0.158	0.239	0.370	0.131	0.151	0.495
Median ventral	0.034	0.082	0.809	0.081	0.157	0.523

CN: cognitively normal; MCI: mild cognitive impairment. All *p* values are FDR adjusted

**Fig. 1:**
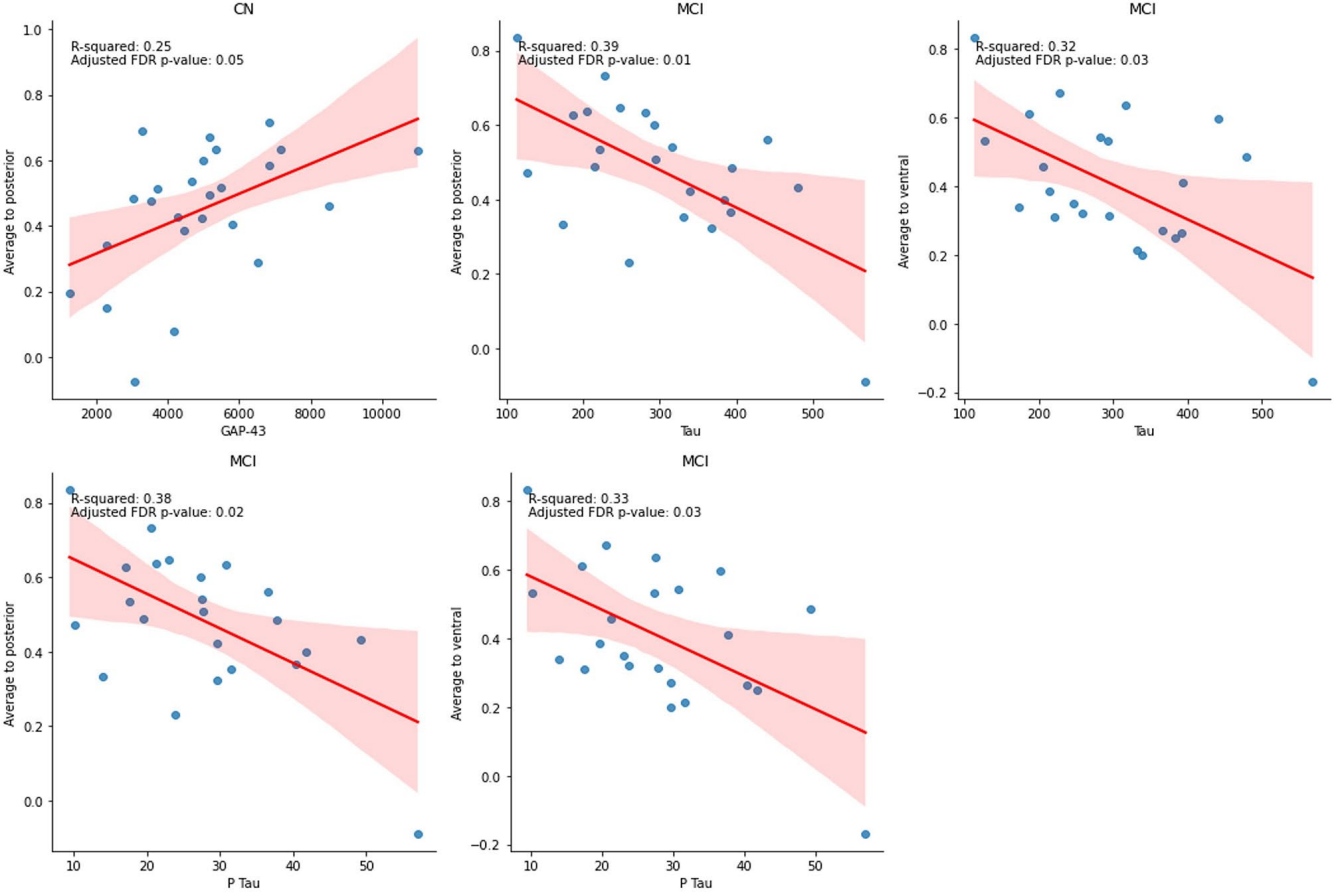
Significant linear regression plots of resting state fMRI and biomarkers. In the CN group, GAP-43 levels positively correlate with the average to posterior measure (R² = 0.25, *p* = 0.05). In the MCI group, T_tau and P_Tau levels negatively correlate with both cognitive measures: T_tau vs. average to posterior (R² = 0.39, *p* = 0.01) and average to ventral (R² = 0.32, *p* = 0.03), and P_Tau vs. average to posterior (R² = 0.38, *p* = 0.02) and average to ventral (R² = 0.33, *p* = 0.03). These R-squared values indicate moderate explanatory power, and the *p*-values show statistical significance after adjustment

Based on linear regression analysis of rs-fMRI measures and tau and P-tau adjusted for age and gender, no significant relationships are found between any specific rs-fMRI measure and tau and P-tau levels in the CN group. However, in the MCI group, both tau and P-tau were found to be inversely associated with both average to posterior and average to ventral, as indicated in Tables 5 and 6. The partial regression plots of all significant linear models are shown in Fig. 1.

**Table 5:** Linear regression analysis of resting state fMRI weighted default mode networks and tau adjusted for age and gender

	CN (*n* = 25)			MCI (*n* = 23)		
	R^2^	Standardized β	*P* value	R^2^	Standardized β	*P* value
anterior to average	0.121	0.331	0.427	0.260	-0.322	0.201
anterior to posterior	0.100	0.256	0.700	0.146	-0.306	0.522
anterior to ventral	0.042	0.155	0.930	0.020	-0.094	0.950
average to posterior	0.080	0.278	0.673	**0.392**	**-0.583**	**0.015**
average to ventral	0.029	0.179	0.899	**0.324**	**-0.544**	**0.033**
posterior to ventral	0.144	0.385	0.261	0.095	-0.095	0.959
Median posterior	0.036	-0.123	0.814	0.142	-0.119	0.602
Median anterior-posterior	0.148	-0.060	0.783	0.006	0.054	0.842
Median average	0.150	-0.235	0.427	0.110	-0.034	0.881
Median ventral	0.035	0.092	0.878	0.220	-0.414	0.181

CN: cognitively normal; MCI: mild cognitive impairment. All *p* values are FDR adjusted. The bold values indicate significance

**Table 6:** Linear regression analysis of resting state fMRI weighted default mode networks and phosphorylated tau adjusted for age and gender

	CN (*n* = 25)			MCI (*n* = 23)		
	R^2^	Standardized β	*P* value	R^2^	Standardized β	*P* value
anterior to average	0.086	0.262	0.744	0.247	-0.296	0.202
anterior to posterior	0.071	0.182	0.691	0.125	-0.266	0.491
anterior to ventral	0.031	0.107	0.924	0.018	-0.082	0.929
average to posterior	0.058	0.229	0.783	**0.382**	**-0.568**	**0.017**
average to ventral	0.036	0.198	0.889	**0.334**	**-0.548**	**0.028**
posterior to ventral	0.133	0.367	0.306	0.090	-0.060	0.991
Median posterior	0.044	-0.157	0.791	0.136	-0.088	0.689
Median anterior-posterior	0.145	-0.015	0.946	0.008	0.073	0.846
Median average	0.145	-0.221	0.469	0.110	-0.031	0.887
Median ventral	0.029	-0.042	0.854	0.271	-0.468	0.086

CN: cognitively normal; MCI: mild cognitive impairment. All *p* values are FDR adjusted. The bold values indicate significance

## Discussion

We aimed to investigate the potential associations between synaptic, Aβ, and tau biomarkers with alterations in the DMN in patients with MCI compared to healthy controls. We found a strong association between brain connectivity in both aDMN and pDMN and the GAP-43 protein measurements in cognitively normal participants. This means that higher connectivity within specific brain regions is associated with elevated levels of GAP-43 protein, suggesting its role in facilitating efficient communication between brain regions. In contrast, individuals with MCI showed disrupted associations between brain connectivity and GAP-43 levels, implying altered neurobiological mechanisms in this population.

GAP-43 is a presynaptic protein located on the cytoplasmic side of the presynaptic membrane [37] and when phosphorylated by protein kinase C, it interacts with other proteins to support axonal outgrowth and vesicular cycling [38]. In vitro hippocampal neurons, GAP-43 is found alongside the axonal marker tau protein [39]. GAP-43 functions in axonal outgrowth, neuroplasticity, and memory formation [40–42] and is highly expressed in various brain regions, including the cerebellum, neocortex, entorhinal cortex, hippocampus, olfactory bulb, and retinal cells, facilitating effective transmission and signal integration and enhancing communication and coordination among different brain regions [43].

Several in vivo studies have formerly suggested that GAP-43 translation may also be triggered by neuronal injury resulting from conditions like stroke, traumatic brain injury, and epilepsy [42, 44–47]. Studies have extensively documented significantly increased levels of GAP-43 protein in the peri-infarct region following experimentally induced cerebral ischemia in rodents [45, 47–50]. These findings suggest that GAP-43 might play a role in neuronal plasticity and preserving the connectivity patterns in the brain.

Although our results suggest that the correlation between CSF GAP-43 levels and brain connectivity among patients with MCI is insignificant, former research has indicated that higher CSF levels of neurogranin and GAP-43 are linked to increased brain metabolism but decreased cortical thickness in brain regions associated with AD. These findings suggest that elevated CSF levels of GAP-43 may be indicative of synaptic dysfunction and neurodegeneration, as reflected by altered brain structure and connectivity in AD-related brain regions [51]. Additionally, multiple studies have shown that elevated baseline levels of CSF GAP-43 are linked to a more aggressive neurodegenerative process, a quicker rate of cognitive decline, and a higher risk of progressing to dementia [12, 17, 52–58]. Since cognitive decline in AD continuum is partly attributed to synaptic dysfunction, GAP-43 is under scrutiny as a marker for both synaptic dysfunction and neurodegeneration.

Based on our findings, a positive relationship between elevated GAP-43 levels in the CSF and enhanced connectivity within the DMN in CN individuals was shown. This correlation underscores the importance of synaptic plasticity in maintaining cognitive health. This means that elevated levels of GAP-43 suggest that the brain is actively engaged in strengthening its neural networks, which is essential for efficient cognitive processing and overall brain health [59, 60]. This enhanced connectivity improves communication between brain regions, facilitating better processing of introspective and self-referential thoughts, and preserving cognitive abilities such as memory, attention, and executive function in cognitively normal individuals. It also provides neuroprotection against age-related decline and neurodegenerative diseases like AD. Additionally, elevated GAP-43 levels can serve as early biomarkers for brain health, identifying individuals at lower risk for cognitive impairments [17, 40, 42, 61].

The DMN is a key brain network active when a person is at rest, involved in self-referential thoughts and processes like introspection and memory [62]. It comprises the aDMN and pDMN. The aDMN includes the medial prefrontal cortex and anterior cingulate cortex, supporting self-reflection, social cognition, and decision-making. The pDMN involves the posterior cingulate cortex and precuneus, essential for memory retrieval, self-awareness, and maintaining consciousness [62–64]. Together, these regions integrate information to maintain a cohesive sense of self.

Based on our findings, there were no significant correlations between Aβ levels and brain connectivity in both groups, suggesting Aβ may not affect brain connectivity in aDMN and pDMN. Nonetheless, former research has suggested that initial Aβ accumulation is linked to reduced connectivity within the DMN and between the DMN and the frontoparietal network [65]. Further, previous studies have also noted a certain overlap between the DMN and the distribution of Aβ fibrils in patients with cognitive impairment and AD [65–72]. For instance, in a recent study utilizing PET and rs-fMRI data, researchers discovered that the limbic and frontoparietal networks exhibit higher annual Aβ accumulation and demonstrate a slower decline in functional connectivity as individuals age. Additionally, the baseline deposition of the amygdala network also experiences a decelerated decline. These findings suggest that the slower decline in functional connectivity observed in healthy aging individuals could potentially serve as a compensatory mechanism for the greater Aβ accumulation [73]. These findings indicate that Aβ exerts a broad influence on functional connectivity, affecting the interplay among various networks and connections, while also correlating with individual cognitive functions.

Several factors including a small sample size, demographic, and methodological differences may explain this discrepancy. These differences may affect the ability to detect associations between Aβ accumulation and functional connectivity in the DMN. It proposes that this relationship is not linear and may be influenced by factors like neuronal activity and metabolic demand. Indeed, high-activity brain cells may lead to increased amyloid beta production or deposit, and metabolic stress, such as energy supply challenges, may also contribute to Aβ accumulation. For instance, animal studies have shown that neuronal activity can enhance Aβ secretion and deposition, suggesting that the increased activity in the DMN may trigger the release and accumulation of Aβ fibrils [74–76]. Another explanation focuses on the high metabolic demand and stress experienced by DMN neurons. These neurons consume a significant amount of energy and frequently undergo fluctuations in activation and deactivation, which may disrupt cellular metabolism and lead to the production and buildup of Aβ [77]. Additionally, Aβ may affect broader networks beyond the DMN, such as the frontoparietal and limbic networks [73, 78], which our focus on the DMN alone might have missed. To reconcile our findings with prior research, it is crucial to consider these factors and conduct future studies with larger, more diverse samples and standardized methodologies to clarify the relationship between Aβ accumulation and brain connectivity.

Most importantly, our findings revealed notable negative correlations within the MCI group between tau and P-tau levels and measures of brain connectivity. These correlations imply that these biomarkers could potentially be linked to neurobiological alterations that play a role in cognitive decline.

Tau pathology in neurodegenerative diseases like AD spectrum triggers a cascade of neuroinflammatory responses in the brain, involving the activation of microglia and astrocytes in response to abnormal tau aggregates [79–83]. When activated, microglia and astrocytes release pro-inflammatory cytokines which per se can exacerbate tau pathology [84–86]. Tau pathology is also associated with dysfunction of the blood-brain barrier [87], allowing peripheral immune cells to infiltrate the brain parenchyma and further contributing to neuroinflammation. Thus, aberrant tau aggregates disrupt brain connectivity, thereby contributing to cognitive decline impairments. Multiple potential pathways have been proposed for how tau pathology can lead to lower connectivity within the DMN. These pathways include the disruption of neuronal communication, synaptic dysfunction, impairment of axonal transport, as well as the disruption of white matter tracts. Tau pathology interferes with normal neuronal functioning, impairs synaptic communication, disrupts axonal transport, and damages white matter tracts, all contributing to the observed decrease in connectivity within the DMN [88, 89]. Indeed, specific regions of the DMN, such as the hippocampus and posterior cingulate cortex, are particularly vulnerable to tau pathology, which further disrupts their connectivity with other DMN regions [89–99].

Lastly, the relatively small sample size of this research was the main limitation which limits the generalizability of the findings. Additionally, while we adjusted for age and gender, we could not account for other demographic factors like socioeconomic status, which could potentially influence the observed associations. Moreover, the study relied on cross-sectional data, which restricts our ability to establish causality or determine the temporal relationships between biomarker levels and fMRI measures. Longitudinal studies would offer more robust evidence regarding the progression of neurobiological changes in cognitive decline. Moreover, the study focused solely on a specific set of biomarkers (GAP-43, Aβ, tau, and P-tau), neglecting other potentially relevant markers of neurodegeneration or neuroinflammation as confounders affecting the results. In short, while the study enhances our comprehension of the neurobiological mechanisms underlying cognitive impairment, its limitations highlight the necessity for larger, longitudinal investigations incorporating a wider array of biomarkers and more refined neuroimaging techniques.

## Conclusion

Focusing on biomarkers such as GAP-43, Aβ, tau, and P-tau, we aimed to understand how these markers correlate with patterns of neural connectivity in aDMN and pDMN. We found that elevated GAP-43 levels in CN individuals are associated with increased connectivity within the aDMN and pDMN, whereas in those with MCI, higher tau and P-tau levels disrupt this connectivity. Surprisingly, we did not find significant correlations between Aβ levels and connectivity measures, suggesting a more complex relationship between Aβ and neural connectivity in aDMN and pDMN in early cognitive decline. These findings elucidate the intricate relationship between biomarkers and brain connectivity within aDMN and pDMN in MCI, highlighting GAP-43 as a potential marker of enhanced connectivity and tau pathology as a disruptor of neural connectivity. Understanding these mechanisms could pave the way for targeted interventions to mitigate cognitive decline in MCI and AD. By addressing limitations in this study, further research is essential to validate these findings and investigate potential therapeutic interventions informed by these mechanisms.

## Data Availability

The data used in this research was obtained from Alzheimer’s Disease Neuroimaging Initiative (ADNI) and is available with permission to all researchers.
